# Polyphenism in social insects: insights from a transcriptome-wide analysis of gene expression in the life stages of the key pollinator, *Bombus terrestris*

**DOI:** 10.1186/1471-2164-12-623

**Published:** 2011-12-20

**Authors:** Thomas J Colgan, James C Carolan, Stephen J Bridgett, Seirian Sumner, Mark L Blaxter, Mark JF Brown

**Affiliations:** 1 Department of Zoology, School of Natural Sciences, Trinity College Dublin, Dublin 2, Ireland; 2 Department of Biology, Callan Building, National University of Ireland Maynooth, Maynooth, Co. Kildare, Ireland; 3 The GenePool Genomics Facility, School of Biological Sciences, University of Edinburgh, West Mains Road, Edinburgh EH9 3JT, UK; 4 Institute of Zoology, Zoological Society of London, Regent's Park, London, NW1 4RY, UK; 5 Institute of Evolutionary Biology, University of Edinburgh, West Mains Road, Edinburgh EH9 3JT, UK; 6 School of Biological Sciences, Royal Holloway, University of London, Egham, Surrey, TW20 0EX, UK

## Abstract

**Background:**

Understanding polyphenism, the ability of a single genome to express multiple morphologically and behaviourally distinct phenotypes, is an important goal for evolutionary and developmental biology. Polyphenism has been key to the evolution of the Hymenoptera, and particularly the social Hymenoptera where the genome of a single species regulates distinct larval stages, sexual dimorphism and physical castes within the female sex. Transcriptomic analyses of social Hymenoptera will therefore provide unique insights into how changes in gene expression underlie such complexity. Here we describe gene expression in individual specimens of the pre-adult stages, sexes and castes of the key pollinator, the buff-tailed bumblebee *Bombus terrestris*.

**Results:**

cDNA was prepared from mRNA from five life cycle stages (one larva, one pupa, one male, one gyne and two workers) and a total of 1,610,742 expressed sequence tags (ESTs) were generated using Roche 454 technology, substantially increasing the sequence data available for this important species. Overlapping ESTs were assembled into 36,354 *B. terrestris *putative transcripts, and functionally annotated. A preliminary assessment of differences in gene expression across non-replicated specimens from the pre-adult stages, castes and sexes was performed using R-STAT analysis. Individual samples from the life cycle stages of the bumblebee differed in the expression of a wide array of genes, including genes involved in amino acid storage, metabolism, immunity and olfaction.

**Conclusions:**

Detailed analyses of immune and olfaction gene expression across phenotypes demonstrated how transcriptomic analyses can inform our understanding of processes central to the biology of *B. terrestris *and the social Hymenoptera in general. For example, examination of immunity-related genes identified high conservation of important immunity pathway components across individual specimens from the life cycle stages while olfactory-related genes exhibited differential expression with a wider repertoire of gene expression within adults, especially sexuals, in comparison to immature stages. As there is an absence of replication across the samples, the results of this study are preliminary but provide a number of candidate genes which may be related to distinct phenotypic stage expression. This comprehensive transcriptome catalogue will provide an important gene discovery resource for directed programmes in ecology, evolution and conservation of a key pollinator.

## Background

A major problem in biology is understanding phenotypic plasticity. Phenotypic plasticity, the ability of a single genotype to express alternative forms of morphology, physiology and behaviour in response to environmental conditions [1], provides the opportunity to study the influences of environment on the genome of an individual or group. Within the natural world, phenotypic plasticity is widespread and has been key to speciation and evolution [1-3]. However, within eusocial species it has resulted in polyphenism, where multiple distinct adult phenotypes result from differential expression of a single genome [4]. Revealing how multiple sets of genes are differentially expressed within the distinct phenotypes of eusocial species offers an unprecedented opportunity to understand the molecular mechanisms related to polyphenisms.

The eusocial Hymenoptera (ants, some bees and wasps) present an excellent system in which to study how gene expression relates to numerous types of polyphenism. Firstly, the social Hymenoptera undergo complete metamorphosis, or holometabolous development, where four morphologically distinct developmental stages (egg, larva, pupa and adult) exist. Holometabolous development is widespread in the superorder Endopterygota and the success of the life history trait is reflected in the high rates of diversification of species that undergo complete metamorphosis [5]. Secondly, they possess caste differentiation within the female sex, where a clear division of labour is evident between two or more physiologically, behaviourally and, in many cases, morphologically distinct phenotypes [6,7]. The reproductive duties of the colony are dominated by a queen while the majority of individuals serve as functionally sterile workers that perform altruistic tasks such as larval feeding, resource foraging, nest maintenance and colony defence [8]. Third, social Hymenoptera display haplodiploid sex determination, with females produced from diploid eggs, while haploid eggs develop into males [9].

Transcriptomic studies have the potential to unveil key gene expression differences that govern central biological processes, such as immunity and olfaction, within and across life cycle stages. A number of studies have been performed in social Hymenoptera to determine which genes are differentially expressed across the adult castes [10-16] and across the sexes [17-19]. These analyses addressed only subsets of the complete transcriptomes of the target species. Recent advances in sequencing technology, such as Roche 454 and Illumina sequencing platforms, generate vastly higher volumes of data [20,21]. As a consequence, these technologies have been used in several studies of non-model species, such as the Granville butterfly [22], the Propertius duskywing and the Anise swallowtail butterflies [23], the migratory locust *Locusta migratoria *[24] and the primitively eusocial wasp, *Polistes metricus *[25]. These studies demonstrate the potential of transcriptomics to provide insights into the expression of polyphenisms in insects, including eusocial species. Thus, the utilisation of transcriptomic tools can significantly improve our knowledge of what genes influence the evolution of polyphenism within eusocial insect species.

The buff-tailed bumblebee *Bombus terrestris*, which is common across Eurasia, is an important ecological pollinator of a wide variety of crops [26,27]. The success of *B. terrestris *as a pollinator is reflected in its increased utilisation in commercial agriculture, a multi-million dollar industry [28]. Caste differentiation in the female sex is evident in *B. terrestris *with a single monandrous queen responsible for the main reproductive duties of the colony while functionally sterile workers perform altruistic tasks. As in all social Hymenoptera, sex determination is haplodiploid. *B. terrestris *has an annual life cycle, where queens overwinter for several months, postmating. Post-hibernation, the overwintered queen establishes a colony in early Spring. She constructs the initial nest, and lays eggs. These hatch after four days into larvae that are completely dependent for feeding from the queen or sister workers for 10 to 14 days [29]. After spinning a cocoon or pupal case, the larva pupates for approximately fourteen days and metamorphoses into the adult [26]. *Bombus *workers display a range of sizes, with size being correlated to function, such as larger bees functioning as foragers [30]. Worker-destined diploid eggs continue to be laid by the queen until a transition point occurs during the colony life cycle, known as the competition phase, where the workers begin to develop functional ovaries and compete with the queen for reproductive output. Workers lay unfertilised male-destined haploid eggs. The initiation of the competition phase coincides with haploid egg laying by the queen while diploid eggs present in the nest may develop into gynes, or virgin queens. Sexuals leave the colony soon after emergence. Virgin queens are mated and subsequently, locate a suitable site to diapause for the duration of the winter months [26].

Two previous transcriptomic studies for *B. terrestris *have been performed. Sadd et al. [31] generated 29,428 expressed sequence tags (ESTs) from the thorax and abdomen of four pooled workers (2 were seven days post-emergence while the second 2 were aged fourteen days post-emergence) using Sanger sequencing technology. More recently, Woodard et al. [32] generated 454 reads from the brains and abdomens of over 50 workers, to include in an analysis of genes central to convergent evolution of eusociality within the bees. While both studies have provided valuable resources for the study of the genomics of *B. terrestris*, they focused on gene expression within workers and as genes may be differentially regulated throughout development, caste or sex, a study incorporating multiple life cycle stages is required to increase our knowledge of gene expression in this important pollinator.

Here we present a deep-sequencing Roche 454 transcriptome study of the pre-adult stages, adult castes, and sexes of *B. terrestris*, based on individual specimens. We identify potentially differentially expressed transcripts related to polyphenism and use differential expression to explore hypotheses for the involvement of two important biological processes, immunity and olfaction, in the life cycle of the bee. These processes were chosen because they represent generally important aspects of animal biology, they are expected to exhibit specific patterns of differential expression in social insects, and they can act as exemplars for the power of the transcriptomic approach. Immune defence against foreign agents would be expected to be heightened in life cycle stages that are more prone to infection, such as larvae, which are housed in a homeostatic nest environment with a high density of nest-mates and consumable resources [33], compared to those that are more protected, such as pupae, which are enclosed in a sealed cocoon. In adults, workers, which have increased exposure to the environment through foraging and increased contact with potentially infected individuals in the colony, and gynes, which need to survive mating and hibernation, would require a heightened immune response to increase longevity compared to the non-social short-lived male [34-36]. Olfaction is a key aspect of animal biology, and in *Bombus *is particularly important for nest-mate recognition and communication [37], resource discrimination [38], subordinate control by the queen [39] and also mate selection [40]. We thus predict that specific olfaction-related genes would be upregulated in the adult stages, for purposes of resource discrimination and mate recognition, while distinct repertoires will be active in pre-adult stages, for caste-development pheromone reception.

## Results

### Sequencing, assembly and assembly validation

**Figure 1:**
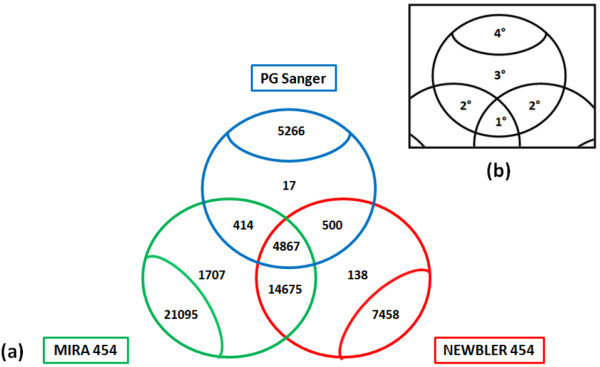
***Bombus terrestris *transcriptome_v1 assembly**. **(a) **Venn diagram depicting the contig contribution to CAP3 second order contigs from the three primary assemblers, MIRA_454 (green), Newbler_454 (red) and PG_Sanger (blue) contigs, consisting of previously available Sanger sequenced ESTs and mRNA available on GenBank. Venn diagram displays overlaps between the first order contig assemblers in CAP3 second order assembly. Numbers in bold in the figure correspond to the number of contigs assembled or unassembled from primary assemblies by CAP3. **(b) **Map of regions of Venn diagrams depicting range in contig credibility: 1° contigs are highly credible, consisting of contributions from all three primary assemblies; 2° contigs are less credible contigs, consisting of contributions from only two primary assemblies; 3° contigs consist of CAP3 contigs assembled from only one primary assembly set; 4° contigs are unassembled contigs from the primary assembly and are considered poorly credible.

Six cDNA preparations were sequenced using the Roche 454 Life Sciences GS FLX Titanium Series sequencer, generating a total of 1,610,742 sequences. After removal of adapters and poly(A) tails, and trimming for base quality, there were 1,560,873 high quality sequences with an average length of 323 bases. Using the method outlined by Kumar and Blaxter [41], these were assembled using two different assemblers, Roche 454's gsAssembler ("Newbler") and MIRA, to generate the first-order assemblies that we will refer to as Newbler_454 and MIRA_454. There were 38,212 contigs in Newbler_454 with a mean length of 650 bases, while MIRA_454 contained 65,786 with a mean length of 668 bases (detailed assembly information is provided in Additional file 1). Using PartiGene, 29,428 *B. terrestris *Sanger ESTs generated by Sadd et al. [31] and 234 *B. terrestris *mRNA sequences (obtained from EMBL nucleotide database) were clustered into 12,337 contigs (hereafter referred to as PG_Sanger). The three first order assemblies (PG_Sanger, Newbler_454 and MIRA_454) were coassembled using CAP3 to generate the BT_transcriptome_v1 contig set. This contained 22,318 contigs with contributions from more than one first order contig, of which 4,867 had contributions from all three first order assemblies, and 33,819 additional contigs containing single first order contigs (Figure 1). As expected, the improved data sampling in terms of depth, life cycle stage, caste and sex resulted in there being an excess (14,675 contigs) of CAP3 contigs with contribution from only Newbler_454 and MIRA_454. We regard those contigs with independent evidence from Sanger and 454 data, and presence in all three primary assemblies as being highly credible. CAP3 contigs with contributions from two or only one primary assembly are likely to be of lower credibility.

We noted that there was still some residual redundancy in BT_transcriptome_v1 contig set, and thus reclustered using the 56,137 second order contigs with PartiGene (removing 1,249 contigs with length of 100 bases or less). This dataset, BT_transcriptome_v2, contained 36,354 contigs. Of these, 25,886 were unchanged from BT_transcriptome_v1, while the remaining 29,210 BT_transcriptome_v1 contigs were clustered into 10,468 BT_transcriptome_v2 contigs. BT_transcriptome_v2 contigs have an average length of 1,102 bases (minimum length = 100 bases, maximum length = 26,105 bases) with an N50 contig size of 1,533 bases. The total span of the BT_transcriptome_v2 contigs is 40MB. Full details of the assembly are provided in Additional file 1.

PG_Sanger contigs that did not coassemble with the 454 primary assemblies were examined for biological credibility. Approximately 75% (22,142) of the *B. terrestris *Sanger sequences were included in contigs with 454 data while in comparison, 862,818 (55% of total high quality screened reads) 454 reads mapped to the PG_Sanger contigs. The remaining 7,519 Sanger sequences clustered into 5,266 contigs, of which 4,091 (77%) were singletons. Thus we conclude that the Sanger-sequencing derived contigs that lack 454 data support are more likely to be rarely expressed genes or technical artefacts. However, AT content was approximately equal for both sets (63.8% for Sanger-only compared to 64% for those that coassembled with 454), suggesting that these isolated contigs do not derive from genomic contamination. In terms of gene content, 43% of the 5,266 Sanger-only contigs had significant (BLASTx E-value < 1e-6) matches to the nonredundant protein database, again suggesting that many have recognisable coding potential and are less likely to be artefacts. Full information on testing of credibility is provided in Additional file 2.

The *B. terrestris *genome project has released a first draft assembly and 95.9% of the BT_transcriptome_v2 contigs (n = 34,861) had high significance megablast matches (E-value < 1e-65) to these data. Woodard et al. [32] generated an assembly of 19,485 *B. terrestris *contigs from Roche 454 data derived from sampling the brains and abdomens of over 50 bees, which were obtained from the TSA database on NCBI. Nearly 75% of these contigs (14,576) matched 9,003 contigs within BT_transcriptome_v2. The contigs unique to the study of Woodard et al. [32] may derive from rare transcripts, or from differences in origin of samples as Woodard et al. [32] generated transcripts from only brains and abdomens of workers while we used whole bodies of several stages.

### Annotation of BT_transcriptome_v2

**Figure 2:**
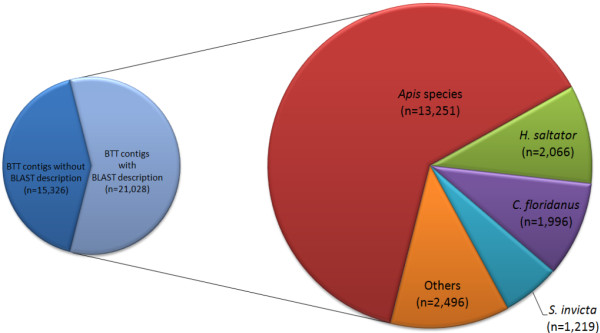
**Pie-chart displaying the species distribution for top BLAST match for BT_transcriptome_v2 contig set against NCBI nr database**. BLASTx, E-value cut-off of 1e-06, against NCBI nonredundant database generated putative matches for 21,028 *B. terrestris *sequences. Species generating the most putative matches were Hymenopteran insects: 13,251 (*Apis species*); 2,066 (*C. floridanus*); 1,996 (*H. saltator*); 1,219 (*S. invicta*); 608 (*Nasonia *species); and 410 (*Bombus *species). Others account for 1,478 putative BLAST matches for BT_transcriptome_v2 contigs.

We compared BT_transcriptome_v2 to the NCBI nr protein database and found significant matches (BLASTx, with E-value cut-off of 1e-06) for 57.8% (n = 21,028) contigs (Figure 2). Of these contigs, 14,268 were assembled from more than one first order assembly. Based on the fact that these contigs were independently assembled using two different algorithms, we may have greater confidence in the biological validity of these 14,268 contigs. The majority of top-scoring matches (20,958 or 57.6%) were to Hymenoptera, including *Apis *species (36.4% of all contigs), the ant species *Harpegnathos saltator *(5.7%), *Camponotus floridanus *(5.5%) and *Solenopsis invicta *(3.4%), the parasitoid wasp *Nasonia *species (1.7%) and *Bombus *species (1.1%) (the proportional representation reflecting the available protein sequence data). We compared the transcriptome assembly to the whole-genome derived proteomes of a set of model Hymenoptera and other insects. Of the 36,354 BT_transcriptome_v2 contigs, 19,744 (54.3%) matched a predicted protein from the honeybee *Apis mellifera *genome, 19,640 (54.0%) matched predicted proteins in the ant *C. floridanus*, and 19,762 (54.4%) matched predicted proteins in the ant *H. saltator*. There were 14,258 (39.2%) matches to the *Drosophila melanogaster *transcriptome. To generate a gene estimate for our BT_transcriptome_v2 contig set, we examined the amount of best BLAST matches (tBLASTx cut-off of 1e-10) between our transcriptome set and the OGS of the honeybee, *A. mellifera*. In total, the BT_transccriptome_v2 contig set matched 9, 217 unique predicted proteins from the latest Apis genome OGS suggesting the potential for an equal number of homologous protein-encoding genes within our transcriptome set. Contigs that had no match to a previously predicted protein may derive from non-coding RNAs, untranslated regions of mRNAs, or from protein coding genes highly diverged in or novel to *B. terrestris*.

Functional annotation classification using the GO, EC and KEGG ontologies using annot8r resulted in the assignment of 533,897 GO terms, 14,345 EC terms and 47,355 KEGG terms to BT_transcriptome_v2 contigs. Approximately 39% (n = 13,996) of contigs were annotated with GO terms. While many other contigs had significant BLAST matches in protein databases, these were to genes that have no GO annotation. GO-Slim analyses are provided in Additional file 3. InterProScan searches were performed generating predicted protein signatures for 24,998 BT_transcriptome_v2 contigs, of which 10,358 contigs received InterProScan (IPR) annotations with information regarding protein family, occurrences of functional domains and repeats. 14,640 contigs had no IPR annotation but were annotated with functional domains.

### Conservation of major biological processes and pathways across Insecta

To assess the completeness of the *B. terrestris *transcriptome represented in our data, we investigated the presence in BT_transcriptome_v2 of genes in conserved developmental and physiological pathways across a set of sequenced insect genomes. For each pathway or system, a set of canonical genes was collated from the proteomes of four species (the eusocial hymenopteran, *A. mellifera*, the solitary hymenopteran, *Nasonia vitripennis*, the holometabolous dipteran, *D. melanogaster*, and the hemimetabolous hemipteran, *Acyrthosiphon pisum*; see Additional file 4) and these were compared to BT_transcriptome_v2 (Table 1). For each species, between 98.5% and 96.7% of the surveyed genes had matches in BT_transcriptome_v2, suggesting that deep sequencing with 454 technology has indeed yielded comprehensive insight into the core regulatory machinery in *B. terrestris*.

**Table 1:** Conservation of biological processes and pathways across Insecta.

Biological Processes and Pathways	*A. mellifera*	*N. vitripennis*	*D. melanogaster*	*A. pisum*
***Development***				
Dorsoventral Axis Formation	21 (22)	10 (10)	63 (65)	17 (17)
Progesterone mediated oocyte maturation	50 (50)	41 (41)	59 (59)	37 (39)
				
***Developmental Signalling Pathways***				
Decapentaplegic	21 (22)	19 (19)	33 (34)	23 (24)
Hedgehog	23 (25)	24 (25)	36 (37)	15 (15)
Notch	16 (17)	18 (18)	31 (32)	18 (19)
Wnt	62 (63)	60 (60)	118 (119)	49 (50)
				
***Metabolism***				
Metabolic Pathways	526 (532)	497 (508)	1118 (1169)	528 (533)
				
***Physiology***				
Circadian rhythm	6 (7)	7 (7)	31 (36)	8 (8)
JAK-STAT	13 (14)	11 (12)	*	17 (17)
MAPK	9 (11)	6 (7)	25 (28)	15 (17)
mTOR	23 (23)	20 (20)	42 (43)	25 (25)
Neuroactive-ligand binding receptor	27 (29)	15 (15)	43 (43)	12 (12)
Phototransduction	26 (28)	24 (24)	45 (46)	17 (17)
Proteasome	35 (35)	35 (35)	50 (50)	45 (45)
				
***Defence***				
Endocytosis	69 (69)	66 (66)	139 (139)	75 (75)
Lysosome	51 (52)	53 (53)	113 (116)	55 (59)
Natural Killer Cell Cytotoxicity	19 (19)	13 (13)	36 (36)	22 (22)
Phagosome	38 (39)	50 (51)	99 (100)	44 (44)
Regulation of Autophagy	12 (12)	11 (11)	18 (18)	14 (14)
				
**Total**	1047	980	2099	1036
(proportion)	97.9%	98.5%	96.7%	98.5%

* Pathway components unavailable

Biological processes and pathways with the number of pathway proteins with significant matches to BT_transcriptome_v2 contigs in the honeybee, *A. mellifera*, the jewel wasp, *N. vitripennis*, the fruitfly, *D. melanogaster*, and the pea aphid, *A. pisum*. The number of reference proteins are given in brackets.

### Life cycle differential expression in *B. terrestris*

**Figure 3:**
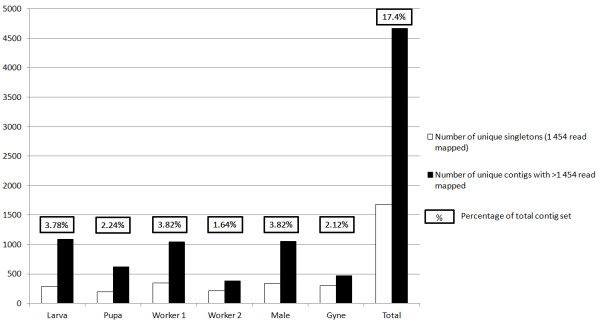
**Contigs unique to *B. terrestris *life cycle stages within the BT_transcriptome_v2 contig set**. Column chart displaying the number of singletons (sequences consisting of 1 454 read) and contigs containing more than 1 454 read generated uniquely from reads contributed from each of the *B. terrestris *libraries within the BT_transcriptome_v2 contig set. The proportion of the stage restricted contigs is provided as a percentage of the total BT_transcriptome_v2 contig set.

We estimated the expression level of each contig by counting 454 reads mapped back to the BT_transcriptome_v2 dataset. The majority (1,441,743 ESTs or 92.4%) were mapped uniquely to 33,875 contigs. 7,635 contigs (22%) received ESTs from all life cycle stages, and these universally expressed genes represented a majority (a mean of 74.5%) of the ESTs from each life cycle stage. There were 1,678 (4.9%) singletons (contigs with a single EST mapping). For 6,341 of the 33,875 contigs expression was detected in only one life cycle stage (Figure 3). The larval and pupal stages had the highest proportion of stage-restricted contigs (~4% each). The adult stages had few stage-restricted contigs (workers: 3,865 [0.75% of total worker reads]; male: 4,182 [1.62% of total]; gyne: 1,449 [0.65% of total]). The two worker samples were very similar, with 95% (worker 1) or 97% (worker 2) of reads mapping to 13,611 shared contigs. Further information regarding the number of unique contigs is provided in Additional file 5.

### R-STAT analysis of differential gene expression amongst life cycle stages and castes

Although our life cycle stage samples are non-replicated, with the exception of the workers (N = 2), to provide a preliminary assessment of potential differential expression across the life cycle stages of the bee, we employed the R-STAT method devised by Stekel et al. [42]. As we did not replicate across life cycle stages, with the exception of the workers, we do not have a full understanding of age or individual variation within the differential expression analysis but operating under the assumption that variation would be minimal between life cycle stages, we examined the following. In total, 2,185 BT_transcriptome_v2 contigs were identified as having high R-values (R-value > 20; following the methods by Stekel et al. [42]). The thirty contigs that had the highest R-values amongst all life cycle stages are detailed in Table 2. The ten genes that best distinguish the different life-stages are provided in Additional file 6.

**Table 2:** R-STAT analysis for the life cycle stages of *B. terrestris*.

BTT_contig	Functional description	Best BLAST Match and GenBank Accession [species]		Read counts per contig							
				
				Larva^a^	Pupa^b^	Worker1^c^	Worker2^d^	Male^e^	Gyne^f^	Total^g^	R-value
BTT05460_1	Alpha-glucosidase	Alpha-glucosidase [*B. ignitus*]	BAI44030.1	110	6	2677	6080	27	3	8903	10101
BTT05364_1	Allergen-related	PREDICTED: similar to CG4409-PA [*A. mellifera*]	XP_001123230.1	5518	1	0	0	0	1	5520	8977
BTT03121_2	Endocuticle structural glycoprotein	Endocuticle structural glycoprotein SgAbd-1 [*C. floridanus*]	EFN60841.1	5208	3	0	0	0	0	5211	8466
BTT05275_2	Hexamerin	Hexamerin [*A. mellifera*]	ABR45904.1	41	45	434	16	55	4884	5475	7619
BTT05433_2	Hexamerin	Hexamerin 110 [*A. mellifera*]	ABU92559.1	283	3384	0	0	0	0	3667	6628
BTT05434_1	Peregrin-like	PREDICTED: similar to CG1845-PA [*A. mellifera*]	XP_395348.2	34	2638	38	21	26	38	2795	5011
BTT05442_1	Hexamerin	Hexamerin 110 [*A. mellifera*]	NP_001094493.1	119	2428	0	0	1	1	2549	4834
BTT05434_2	Beta-ureidopropionase	PREDICTED: similar to beta-ureidopropionase isoform 1 [*A. mellifera*]	XP_392773.2	17	2489	34	20	32	18	2610	4805
BTT35255_1	Hexamerin	Hexamerin 110 [*A. mellifera*]	ABU92559.1	346	2376	0	0	0	0	2722	4551
BTT20945_1	Hexamerin	Hexamerin 110 [*A. mellifera*]	BAI82215.1	147	2304	0	1	0	0	2452	4547
BTT17751_1	Short-chain dehydrogenase/Reductase	Short-chain dehydrogenase/reductase [*A. mellifera*]	NP_001011620.1	0	1760	0	0	0	0	1760	3708
BTT05366_1	Endocuticle structural glycoprotein	PREDICTED: similar to CG7658-PA [*A. mellifera*]	XP_001120498.1	2153	0	0	0	0	0	2153	3489
BTT23482_1	Hexamerin	Hexamerin [*A. mellifera*]	ABR45905.1	1	1633	0	1	0	1	1636	3423
BTT35822_1	Hexamerin	Hexamerin [*A. mellifera*]	ABR45905.1	0	1585	0	0	0	0	1585	3337
BTT18911_1	Hexamerin	Hexamerin 70b [*A. mellifera*]	NP_001011600.1	2947	15	337	88	99	177	3663	3289
BTT07410_1	Vitellogenin	PREDICTED: hypothetical protein [*A. mellifera*]	XP_001121939.1	0	1512	0	0	0	1	1513	3177
BTT05437_1	Hexamerin	Hexamerin 70c [*A. mellifera*]	NP_001092187.1	1	1496	0	0	0	0	1497	3142
BTT20899_3	Sallimus	PREDICTED: similar to sallimus CG1915-PC, isoform C [*A. mellifera*]	XP_001121572.1	7	0	1176	1922	16	305	3426	2960
BTT20391_1	Bomboilitin	Bombolitin [*B. ignitus*]	ACY09649.1	5	1	1677	1691	9	837	4220	2944
BTT21092_1	Alpha-glucosidase	Alpha-glucosidase [*B. ignitus*]	BAI44030.1	31	1	766	1685	3	0	2486	2822
BTT05276_2	Allergen-related	Allergen-related G12 [*H. saltator*]	EFN86980.1	0	0	1948	819	2267	458	5492	2658
BTT38955_1	Beta-ureidopropionase	PREDICTED: similar to beta-ureidopropionase isoform 1 [*A. mellifera*]	XP_392773.2	8	1190	11	7	13	10	1239	2316
BTT24170_1	Hymenoptaecin	Hymenoptaecin [*B. ignitus*]	ACA04899.1	1	22	0	0	1345	1	1369	2227
BTT05294_1	Cytochrome P450	Cytochrome P450 4g15 [*H. saltator*]	EFN85148.1	106	29	1568	2115	576	762	5156	2201
BTT20843_1	Larval cuticle protein	PREDICTED: similar to CG30045-PA [*A. mellifera*]	XP_001120541.1	1355	0	0	0	0	0	1355	2187
BTT32853_1	Hexamerin	Hexamerin [*A. mellifera*]	ABR45905.1	1	943	0	0	0	0	944	1971
BTT24170_2	Hymenoptaecin	Hymenoptaecin [*B. ignitus*]	ACA04899.1	0	15	1	0	1171	0	1187	1948
BTT33135_1	Cytochrome P450	Cytochrome P450 4G11 [*A. mellifera*]	NP_001035323.1	84	10	1990	1472	541	827	4924	1944
BTT12993_1	Carbonic anhydrase	Hypothetical protein SINV_10521 [*S. invicta*]	EFZ18639.1	1732	8	134	49	252	30	2205	1937
BTT05272_1	Hypothetical protein	PREDICTED: similar to CG3246-PA [*A. mellifera*]	XP_395658.3	7	41	1792	871	1453	1399	5563	1916

a = Total number of larva ESTs (n = 286501); b = Total number of pupa ESTs (n = 162608); c = Total number of worker1 ESTs (n = 318664); d = Total number of worker2 ESTs (n = 194969); e = Total number of male ESTs (n = 257318); f = Total number of gyne ESTs (n = 221683); g = Total number of ESTs (n = 1441743).

Mapped read contributions from each sequenced life cycle stage were analyzed using R-STAT to identify contigs with potential differential expression. Read counts are provided for each life cycle stage with the corresponding R-value. BT_transcriptome_v2 contigs (BTT_contig) reference, functional description, best NCBI nonredundant (nr) BLAST match description and GenBank accession are provided for each contig.

### Gene expression within the workers

The contig with the highest R-value (BTT05460_1; R-value = 10,101) was identified as alpha-glucosidase, with elevated expression in both workers (Table 2; Additional file 6, Tables S6c and S6d). The workers also had elevated expression of genes involved in flight (sallimus; BTT20899_3); defence (bombolitin; BTT20391_1); metabolism (two contigs, BTT05294_1 and BTT33135_1, matching cytochrome P450 proteins); and a protein with domain matches to both haemolymph juvenile hormone binding and mite allergen group-7 (BTT05272_1).

Examination of the ten genes that best distinguish the worker caste (based on genes highly expressed in both worker samples) identified genes involved in metabolism, chitin binding and defence (see Additional file 6: Tables S6c and S6d)). Both workers exhibited high expression of genes involved in metabolism, such as oxidation of organic compounds (three contigs matching three cytochrome P450 proteins (BTT22253_1, BTT24074_1 and BTT22199_1)), and glucose breakdown, (glyceraldehyde-3-phosphate dehydrogenase (BTT00029_1) and glucose oxidase (BTT20590_1)). A lipase (BTT15820_1), involved in the breakdown of lipids essential for periods of high activity, was also highly expressed in the workers. Two contigs (BTT05313_2 and BTT35235_1) matched chitin-binding, peritrophin-like proteins. Two worker-upregulated contigs matched genes implicated in immune defence: allergen-related G12 protein (BTT05276_1) and bombolitin (BTT05263_2). Other contigs highly differentially expressed in workers included diapause-related protein 41 (BTT05480_1; physiological function unknown), and two contigs matching hypothetical proteins from the ant *C. floridanus *(BTT20743_1) and the honeybee (BTT05577_1). BTT05480_1 and BTT20743_1 have both transmembrane and signal peptide domains suggesting a cell-surface or membrane-bound location.

### Gene expression in the gyne

The top ten differentially expressed genes included elevated expression in the gyne of an amino acid storage protein, hexamerin (BTT05275_2) (see Table 2). Within the ten genes that best distinguish the gyne (see Additional file 6: Table S6f), five contigs matched storage proteins: four hexamerins (BTT05275_2, BTT36615_1, BTT05275_1 and BTT05277_1) and one arylphorin (BTT05260_1). Apart from genes involved in storage, one contig (BTT00028_1) matched acyl-CoA delta-9 desaturase, a protein involved in fatty acid biosynthesis, another a chitin-binding peritrophin (BTT05289_2) and a third crooked, a protein involved in maintenance of tracheal tube structure (BTT18720_1). Two contigs had matches to hypothetical proteins in the ant *Brachymyrmex patagonicus *(BTT07422_1) and the ant *C. floridanus *(BTT20597_1). BTT07422_1 was annotated with a predicted signal peptide domain while BTT20597_1 had predicted transmembrane, signal peptide and fibronectin-like 1 domains.

### Gene expression in the male

The male sample had a high expression of genes involved in immunity (see Table 2). The male contributed a high proportion of ESTs to a contig similar to the allergen protein G12 (BTT05276_2) and also exhibited high expression of two contigs encoding antimicrobial peptides identified as hymenoptaecins (BTT24170_1 and BTT24170_2). Within the top ten genes that distinguished the male (see Additional file 6, Table S6e) a third hymenoptaecin contig was identified (BTT36277_1). Immunity aside, the male had high expression of genes involved in metabolism (i.e. sentrin-like protease; BTT06274_2) and a serine carboxypeptidase (BTT05501_1), flight (i.e. the muscle protein titin; BTT05775_1) and cuticle formation (i.e. the peritrophin like gene BTT05289_1). Two contigs of unknown function were also overexpressed in the male. One had a predicted fibronectin-like domain (BTT00570_1) while the second contig had transmembrane and signal peptide domains (BTT09205_1).

### Gene expression in the larva

The pre-adult stages accounted for 19 of the top thirty highest R-value annotated contigs (6 from the larva and 13 from the pupa). The larva (Table 2) had elevated expression of proteins involved in cuticle formation (BTT03121_2, BTT05366_1, BTT20843_1 and BTT20746_1), and amino acid storage (hexamerin 70b BTT18911_1). A member of the cytochrome P450 superfamily (BTT20966_1) was also highly expressed in the larva. Within the larva we identified elevated expression of additional genes involved in development and cuticle formation (Additional file 6, Table S6a). The enzyme, carbonic anhydrase, which is involved in metabolism, was highly expressed (BTT12993_1). Two functionally unidentified contigs were also overexpressed, one of which (BTT35627_1) had predicted transmembrane and signal peptide domains.

### Gene expression within the pupa

In contrast to the high expression of cuticular and structural proteins in the larva, the pupa had a higher expression of a diversity of amino acid storage proteins (Table 2; Additional file 6, Table S6b). Of the amino acid groups, seven contigs matched hexamerins: four to hexamerin 110 (BTT05433_2, BTT05442_1, BTT35255_1 and BTT20945_1), one to hexamerin 70c (BTT05437_1) and two to an unclassified hexamerin (BTT23482_1 and BTT35822_1). There was a high pupal EST contribution to a contig matching a peregrin-like protein, which has a role in dorsal/ventral axon guidance, an important biological process during transformation from larva to adult. Of the three contigs putatively matching metabolic enzymes, two contigs (BTT05434_2 and BTT38955_1) matched beta-ureidopropionase, an enzyme involved in metabolism of pyrimidine and beta-alanine, while one matched a short-chain dehydrogenase-reductase (BTT17751_1). Vitellogenin, which has a role in lipid transport, was highly expressed in the pupa (BTT07410_1) in comparison to the larva.

### Differential expression of immunity-related genes across the *B. terrestris *life cycle

**Figure 4:**
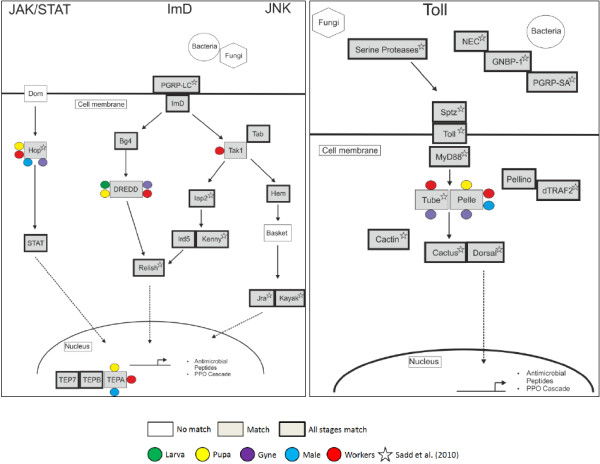
**The immune system in *Bombus terrestris***. The *B. terrestris *transcriptome data were screened for the presence of components of the major immune signalling pathways using reciprocal tBLASTx searches between BT_transcriptome_v2 and *A. mellifera *sequences. Some components had stage limited expression, as indicated by the coloured circles. Pathway components also identified by Sadd *et al*. [31] are indicated by star symbols.

The development and expression of the immune response in *B. terrestris *is particularly interesting (see Introduction). We identified *B. terrestris *homologues of the four major *A. mellifera *immune signalling pathways (*Toll*, Immune deficiency (ImD), *Janus *kinase and signal transducer and activator of transcription (JAK-STAT), and JNK immune signalling) in BT_transcriptome_v2 contigs, and analysed these for expression differences among life cycle stages (Figure 4). Of the *Toll *pathway components, expression was uniform across life cycle stages apart from two signalling components, *Tube *and *Pelle*. Neither was expressed in the pupa, while *Tube *was only detected in the female adult stages (workers and gynes). For the ImD, JNK, and JAK-STAT signalling pathways, two components were not present in BT_transcriptome_v2: the transmembrane receptor *Domeless*, and the transcription factor for the JNK pathway, JNK MAP Kinase *basket*. Components of these three pathways were detectably expressed in all stages, but TEPA, DREDD, and TGF-β activated kinase 1 (TAK1) were only expressed in workers. Sadd et al. [31] also enumerated pathway component expression in workers, but we identified more complete coverage of expected pathways, including a complete *Toll *signalling pathway.

Ten immunity-related effector genes were differentially expressed across the sequenced specimens (Table 3). Nine of these were expressed in all life cycle stages (the antimicrobial peptide (AMP) abaecin was absent from the larva). Phenoloxidase subunit A3 (BTT08527_1), which functions in the production of melanin and other polyphenic compounds for both cuticle biosynthesis and immune encapsulation, had highest expression in the pupa. A C-type lectin (BTT07416_1) and a gram-negative binding protein (BTT09196_1), which both have roles in immune detection of bacteria, had elevated expression in the larva, while the gram-positive binding peptidoglycan recognition protein (PGRP) SA was highly expressed in the gyne (BTT21344_1). Transferrin, an iron chelator that impacts on the survival of bacteria within a host, matched 22 contigs with differential expression, of which 20 contigs had high EST contributions in the male while two contigs had high EST contribution from the gyne (BTT35862_1) and the larva (BTT35539_1), respectively (Table 3). The male had elevated expression of the AMPs abaecin, apidaecin, hymenoptaecin and defensin 1 (Table 3: Anti-microbial peptides). Several distinct contigs encoding the *Bombus*-specific AMP bombolitin (BTT20391_1, BTT34958_1, BTT35608_1 and BTT40712_1), a constituent of venom, showed elevated expression in the female adults, especially the workers.

**Table 3:** Potential differential expression of immunity-related genes across the life cycle stages of *B. terrestris*.

BTT_contig	Best BLAST Match and GenBank Accession [species]		Read counts per contig							
	
	*Immune-related Genes*		Larva^a^	Pupa^b^	Worker1 ^c^	Worker2 ^d^	Male ^e^	Gyne ^f^	Total ^g^	R-value
BTT08527_1	Phenoloxidase subunit A3 [*A. mellifera*]	NP_001011627.1	59	**78**	4	1	3	6	151	123
BTT24384_1	Transferrin [*A. mellifera*]	AAO39761.1	38	58	106	68	**530**	152	952	395
BTT35190_1	Transferrin [*A. mellifera*]	AAO39761.1	1	0	7	0	**27**	4	39	25
BTT35213_1	Transferrin [*A. mellifera*]	AAO39761.1	94	83	199	96	**292**	238	1002	95
BTT35261_1	Transferrin [*A. mellifera*]	AAO39761.1	12	9	86	13	**148**	64	332	113
BTT35441_1	Transferrin [*A. mellifera*]	AAO39761.1	7	3	13	17	**49**	20	109	29
BTT35539_1	Transferrin [*A. mellifera*]	AAO39761.1	**34**	0	15	30	4	17	100	28
BTT35592_1	Transferrin [*A. mellifera*]	AAO39761.1	9	0	13	3	**74**	20	119	66
BTT35613_1	Transferrin [*A. mellifera*]	AAO39761.1	9	6	28	16	**64**	34	157	35
BTT35644_1	Transferrin [*A. mellifera*]	AAO39761.1	10	14	55	9	**117**	53	258	82
BTT35679_1	Transferrin [*A. mellifera*]	AAO39761.1	0	0	60	3	**119**	37	219	134
BTT35708_1	Transferrin [*A. mellifera*]	AAO39761.1	0	0	8	0	**24**	3	35	23
BTT35750_1	Transferrin [*A. mellifera*]	AAO39761.1	4	5	35	6	**55**	27	132	39
BTT35862_1	Transferrin [*A. mellifera*]	AAO39761.1	0	0	15	0	0	**150**	165	236
BTT35895_1	Transferrin [*A. mellifera*]	AAO39761.1	0	0	10	0	**31**	10	51	31
BTT35931_1	Transferrin [*A. mellifera*]	AAO39761.1	13	0	18	1	**35**	17	84	24
BTT35947_1	Transferrin [*A. mellifera*]	AAO39761.1	0	6	8	1	**37**	8	60	33
										
BTT07416_1	PREDICTED: similar to CG3244-PA, partial [*A. mellifera*]	XP_624536.1	**375**	9	6	4	7	38	439	463
										
BTT21344_1	Peptidoglycan-recognition protein SA [*A. mellifera*]	NP_001157187.1	56	159	116	22	72	**169**	594	121
BTT09196_1	Gram-negative bacteria-binding protein 1-2 [*A. mellifera*]	NP_001157186.1	**141**	10	90	57	86	26	410	56
										
	**Anti-microbial Peptides**									
BTT39615_1	Abaecin [*A. mellifera*]	AAA67442.1	0	0	0	0	**24**	0	24	30
BTT08491_1	Abaecin [*A. mellifera*]	AAA67442.1	0	0	0	0	**45**	0	45	64
BTT20802_1	Abaecin [*A. mellifera*]	AAA67442.1	0	5	0	0	**122**	6	133	174
BTT37089_1	Abaecin [*A. mellifera*]	AAA67442.1	0	0	2	1	**57**	6	66	71
BTT38306_1	Abaecin [*A. mellifera*]	AAA67442.1	0	0	1	1	**31**	0	33	38
										
BTT05666_1	Apidaecin [*A. mellifera*]	CAA51168.1	3	7	1	3	**55**	3	72	59
BTT20828_1	Apidaecin [*A. mellifera*]	CAA51168.1	9	33	7	9	**128**	16	202	123
BTT35749_1	Apidaecin [*A. mellifera*]	CAA51168.1	2	2	14	24	8	**29**	79	27
BTT38204_1	Apidaecin [*A. mellifera*]	CAA51168.1	0	4	1	2	**25**	0	32	27
BTT41196_1	Apidaecin [*A. mellifera*]	CAA51168.1	0	11	2	3	**137**	7	160	180
BTT41985_1	Apidaecin [*A. mellifera*]	CAA51168.1	4	13	3	4	**81**	2	107	90
										
BTT20391_1	Bombolitin [*B. ignitus*]	ACY09649.1	5	1	1677	**1691**	9	837	4220	2944
BTT34958_1	Bombolitin [*B. ignitus*]	ACY09649.1	1	0	**49**	18	1	15	84	46
BTT35608_1	Bombolitin [*B. ignitus*]	ACY09649.1	0	0	**201**	98	0	180	479	317
BTT40712_1	Bombolitin [*B. ignitus*]	ACY09649.1	0	0	**341**	148	1	133	623	422
										
BTT10405_1	Defensin 1 [*B. terrestris*]	ADB29129.1	2	2	0	0	**85**	0	89	122
BTT31016_1	Defensin 1 [*B. terrestris*]	ADB29129.1	0	0	0	0	**179**	0	179	289
BTT41393_1	Defensin 1 [*B. terrestris*]	ADB29129.1	3	0	3	3	**25**	1	35	21
BTT42034_1	Defensin 1 [*B. terrestris*]	ADB29129.1	0	2	3	3	**47**	0	55	56
										
BTT24170_1	Hymenoptaecin [*A. mellifera*]	AAA67444.1	1	22	0	0	**1345**	1	1369	2226
BTT24170_2	Hymenoptaecin [*A. mellifera*]	AAA67444.1	0	15	1	0	**1171**	0	1187	1948
BTT36277_1	Hymenoptaecin [*A. mellifera*]	AAA67444.1	0	8	3	0	**882**	0	893	1460
BTT37570_1	Hymenoptaecin [*A. mellifera*]	AAA67444.1	0	0	0	0	**41**	0	41	57
BTT41217_1	Hymenoptaecin [*A. mellifera*]	AAA67444.1	0	0	0	0	**70**	0	70	105
BTT18071_1	Hymenoptaecin [*A. mellifera*]	AAA67444.1	0	1	0	0	**27**	0	28	34
BTT35620_1	Hymenoptaecin [*A. mellifera*]	AAA67444.1	0	0	**40**	10	13	15	78	30
BTT36443_1	Hymenoptaecin [*A. mellifera*]	AAA67444.1	12	34	17	10	**47**	38	158	29
BTT37051_1	Hymenoptaecin [*A. mellifera*]	AAA67444.1	0	0	0	0	**183**	0	183	296
BTT41185_1	Hymenoptaecin [*A. mellifera*]	AAA67444.1	0	6	0	0	**64**	0	70	90
BTT42611_1	Hymenoptaecin [*A. mellifera*]	AAA67444.1	0	0	0	0	**20**	0	20	24

a = Total number of larva ESTs (n = 286501); b = Total number of pupa ESTs (n = 162608); c = Total number of worker1 ESTs (n = 318664); d = Total number of worker2 ESTs (n = 194969); e = Total number of male ESTs (n = 257318); f = Total number of gyne ESTs (n = 221683); g = Total number of ESTs (n = 1441743).

Immunity-related genes were identified in BT_transcriptome_v2 and are shown with read counts for each life cycle stage. The highest amount of ESTs per contig is highlighted in bold.

### Differential expression of olfaction genes in *B. terrestris*

**Figure 5:**
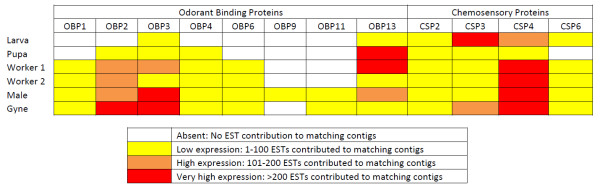
**Olfaction in bumblebees**. BT_transcriptome_v2 contigs were screened for the presence of olfaction-related genes, odorant-binding proteins (OBPs) and chemosensory proteins (CSPs). Some genes had stage-specific expression as indicated by the colour scheme.

We expected that olfaction would differ among castes and life cycle stages because of their differing needs for, and responses to, social and other cues. We examined the presence and differential expression of olfaction-related genes, namely the odorant-binding proteins (OBPs) and the chemosensory proteins (CSPs) (Table 4). Both larva and pupa had low expression of OBPs, with the exception of high expression of OBP13 in the pupa (Table 4 and Figure 5). In addition, OBP2 (BTT27418_1, BTT42039_1) OBP3 (BTT35749_1) and OBP4 (BTT41749_1), OBP13 (BTT26653_1) were expressed in the pupa while OBP3 and OBP13 were only expressed in the larva (Figure 5). Four CSPs, namely CSP2 (BTT00638_1), CSP3 (BTT19102_1), CSP4 (BTT17558_1, BTT26377_1) and CSP6 (BTT35682_1), were expressed in the larva with particularly high expression of CSP3 (BTT19102_1) and heightened expression of CSP4 in comparison to the pupa (BTT34298_1). The pupa only expressed three CSPs (CSP2, CSP3 and CSP4).

**Table 4:** Potential differential expression of olfaction-related genes in the life cycle stages of *B. terrestris*.

BTT_contig	Best BLAST Match and GenBank Accession [species]		Read counts per contig							
	
	*Odorant binding proteins*		Larva^a^	Pupa^b^	Worker1^c^	Worker2^d^	Male^e^	Gyne^f^	Total^g^	R-value
BTT08587_1	OBP 2 [*A. mellifera*]	NP_001011591.1	0	0	129	83	97	**172**	481	193
BTT11797_1	OBP 2 [*A. mellifera*]	NP_001011591.1	0	0	19	14	**40**	11	84	35
BTT20806_1	OBP 2 [*A. mellifera*]	NP_001011591.1	1	0	63	**109**	86	61	320	129
BTT40878_1	OBP 2 [*A. mellifera*]	NP_001011591.1	0	0	11	3	**19**	18	51	20
										
BTT05623_1	OBP 3 [*A. mellifera*]	NP_001035311.1	0	0	32	11	**110**	77	230	134
BTT20849_1	OBP 3 [*A. mellifera*]	NP_001035311.1	0	0	56	32	46	**100**	234	103
										
BTT16540_1	OBP 13[*A. mellifera*]	NP_001035314.1	0	0	0	0	**31**	0	31	41
BTT20296_1	OBP13 [*A. mellifera*]	ABD92645.1	93	**776**	116	22	22	91	1120	1045
BTT26653_1	OBP13 [*A. mellifera*]	ABD92645.1	0	1	0	0	**47**	0	48	66
										
	***Chemosensory Proteins***									
BTT00638_1	CSP 2 [*A. mellifera*]	NP_001071278.1	8	**30**	7	6	19	5	75	23
BTT19102_1	CSP 3 precursor [*A. mellifera*]	NP_001011583.1	**467**	16	56	19	64	126	748	385
BTT17558_1	CSP 4 [*A. mellifera*]	NP_001071282.1	34	20	53	**115**	66	31	319	57
BTT20849_1	CSP 4 [*A. mellifera*]	NP_001071282.1	0	0	56	32	46	**100**	234	103
BTT26377_1	CSP 4 [*A. mellifera*]	NP_001071282.1	15	15	68	**69**	67	43	277	38
BTT34298_1	CSP 4 [*A. mellifera*]	NP_001071282.1	**45**	0	3	3	8	4	63	39
BTT39167_1	CSP 4 [*A. mellifera*]	NP_001071282.1	11	8	**52**	39	37	33	180	22
BTT35682_1	CSP 6 [*A. mellifera*]	NP_001071287.1	1	0	37	16	**99**	25	178	92

a = Total number of larva ESTs (n = 286501); b = Total number of pupa ESTs (n = 162608); c = Total number of worker1 ESTs (n = 318664); d = Total number of worker2 ESTs (n = 194969); e = Total number of male ESTs (n = 257318); f = Total number of gyne ESTs (n = 221683); g = Total number of ESTs (n = 1441743).

Potential odorant-binding proteins and chemosensory proteins in the BT_transcriptome_v2 contigs are shown, with read counts for each of the life cycle stages. The highest amount of ESTs per contig are highlighted in bold.

The adult stages had a heightened expression of OBP2 and OBP3 in comparison to the larval and pupal stages (Table 4). Contigs identified as OBP2 (BTT08587_1; BTT11797_1, BTT20806_1 and BTT40878_1) and OBP3 (BTT05623_1 and BTT20849_1) had elevated expression in the adult stages in comparison to the larva, and were not expressed in pupa. OBP13-like proteins were highly expressed in the pupa (BTT20296_1) and the male (BTT16540_1; BTT26653_1). In addition, OBP1 and OBP6 were detected in all adult stages but in neither the larva nor the pupa. OBP11 was only expressed in the sexuals (male and gyne) while OBP9 was only expressed in the male. Four CSPs expressed in the larva were expressed in the adult stages. There was an elevated expression of CSP4 in the female adults in comparison to the male.

## Discussion

We have used Roche 454 deep sequencing to define and compare the transcriptomes expressed by life cycle stages and castes of *B. terrestris*. Together, these data form a comprehensive gene catalogue for this ecologically and economically important species. The *de novo *assembly had high contiguity, with a mean contig length of 1,102 bases. The G+C content for the contigs was 36%, which is similar to the worker-sequencing results of Sadd et al. [31]. The majority of contigs (approximately 58%) had significant BLAST matches to previously described proteins. The remaining 42% may derive from untranslated regions of unassembled mRNAs, noncoding RNAs or transcriptional noise (retained introns and similar), or, more interestingly, from *B. terrestris *genes that are either novel or have diverged significantly from any sequenced relatives. Comparison of the *B. terrestris *transcriptome against the emerging *B. terrestris *genome sequence identified matches for 95.9% of the contigs, and we expect that our data will be of utility in annotation efforts for this genome (which is currently being carried out by the Bombus sequencing consortium; K. Worley, P. Schmid-Hempel, G. Robinson, pers.comm.). Separate sequencing of individual life stages permitted the identification of potentially differentially expressed genes across the life cycle stages using R-STAT, and identified potentially important candidate genes underpinning stage- and caste-specific phenotypes. While we drew samples from two natal colonies, comparison of expression between the workers from the two colonies demonstrated significant similarity, justifying an overall comparison of pre-adult stages and adult stages.

We used R-STAT analyses to identify a list of potentially differentially expressed contigs, in order to provide direction for future studies examining gene expression within or across life cycle stages of *B. terrestris*. However, some data already exist in support of this approach. In a previous study by Pereboom et al. [11], northern blot analyses examined expression differences between whole bodied larvae and adults (queen and worker stages) and identified three genes (endocuticle structural glycoprotein; hexamerin 70b; and 60S acidic ribosomal protein P2) that were more highly expressed in larvae than in adults, where expression was absent. These genes match contigs generated from our transcriptomic R-STAT analyses that show the same larva-adult expression pattern, providing independent support for the biological validity of the results discussed below.

### Differential gene expression linked to developmental processes

We identified a number of key differences in genes implicated in developmental processes across the different life stages. For example, the larval data showed high expression of genes involved in cuticle biogenesis. As the larval stage represents a period of feeding and growth, undergoing four developmental moults over a fourteen day period, this upregulation of cuticular proteins and endocuticle structural glycoproteins is expected. Other contigs identified in the transcriptome with similar expression profiles are thus likely candidates for genes with similar stage-specific roles. For example, a short-chain dehydrogenase/reductase (SDR) had elevated expression in the pupa. SDRs function in hormone and steroid metabolism [43], and in social insects, may be involved in stage and caste differentiation, for example through binding juvenile hormone. An SDR was demonstrated to be differentially expressed at the mRNA level in the ovaries in developing larvae of honeybee workers and queens, and SDR gene expression was higher in the ovaries of worker larvae in comparison to queen larvae resulting in possible inhibition of ovary development [44]. The likely co-orthologues found in our study of the *A. mellifera *SDR analysed by Guidugli et al. [44] were derived solely from pupal reads, while Guidugli et al. [44] reported expression being highest in final stage honeybee larvae. Thus, we propose that these SDRs are candidate genes for further investigation into their roles in bumblebee caste determination.

### Amino-acid storage protein expression across the bumblebee life cycle

Hexamerins are amino acid storage proteins, related to tyrosinases [45], used during non-feeding periods of adult development to provide amino acids and energy [46]. Hexamerins also have roles in the binding of juvenile hormone during insect development, impacting on growth regulation within the larval stage [47-49]. A number of studies [50-53] have demonstrated differential expression of hexamerin proteins (hexamerin 70a, hexamerin 70b, hexamerin 70c and hexamerin 110) at different stages of development amongst the sexes and castes in the honeybee. In *B. terrestris*, the pupal sample had elevated expression of hexamerin 110-like genes and a hexamerin 70 homologue, while the larva had elevated expression of distinct hexamerin 70-like genes, and the gyne elevated expression of four additional hexamerin 70-like genes. Thus while only four hexamerin-encoding genes were identified in *A. mellifera *[53], we have identified potentially ten in *B. terrestris *that show differential expression between life cycle stages and castes, suggesting that these proteins may play complex roles in bumblebee development.

Why would gynes express high levels of amino acid storage proteins? Honeybee virgin queens have a higher expression of hexamerins in comparison to workers, with the hexamerins functioning in gonad development [53], whilst in the wasp, *P. metricus*, developing gynes have higher quantities of Hex 1 than workers [54]. Studies on hexamerins in other social insects, such as ants and termites, have identified a correlation between depletion of hexamerins within queens and colony formation [55,56]. Therefore, this potentially high expression of hexamerins would be important for a *B. terrestris *gyne from a colony formation viewpoint. However, as *B. terrestris *queens undergo a prolonged hibernation after mating, amino acid storage proteins may be important for maintaining functional operation of crucial biological processes during a period of intense stress. As hibernation is a key stage in the life cycle of bumblebees, many species of which are in drastic worldwide decline [57], our results provide direction for future work to analyse the mechanisms behind successful hibernation in these insects.

### Genes involved in adult behaviour and physiology

Workers had elevated expression of enzymes, such as alpha-glucosidase and a muscle-specific lipase, that would be important for worker task completion. Alpha-glucosidase is involved in carbohydrate metabolism and utilised by foraging honeybees to metabolise nectar into fructose and glucose [58], while the muscle lipase is important for breaking down lipids during periods of high activity [59]. Interestingly, in our study both enzymes were expressed at an early stage in the adult workers' life (only 72 hours old), in contrast to their temporal pattern of expression in *A. mellifera *workers. Honeybee workers demonstrate a strict temporal polyethism, where younger workers perform nursing duties while older workers forage [60], and it is these foraging workers that exhibit higher expression of these enzymes. In comparison, there is no strong age-dependent division of labour within bumblebees [61]. Alloethism within bumblebee workers has been correlated with size, with studies identifying larger workers performing foraging tasks while smaller workers perform in-nest functions, although this division of labour can change depending on the requirements of the colony [30]. Thus future work might focus on size- *and *age-related differences in gene expression between *B. terrestris *workers in relation to their subroles within the colony.

Males are underrepresented in genomic studies in social insects as the emphasis has been almost exclusively on females. In the current study, the male had elevated expression of titin, a muscle protein, expressed in the insect flight muscles [62]. As the male bumblebee requires flight for foraging, patrolling and indeed mating, high expression of flight-specific muscles would be required. In relation to behaviour, the male had a high expression of a neuroparsin, queen brain selective protein 1, which has been suggested to function in caste determination during honeybee development through manipulation of the insect insulin-like pathway [63]. However, neuroparsins have been suggested to play roles in a wide variety of functions, including reproduction [64]. Therefore, a neuroparsin-like protein in the bumblebee may have male-specific expression in relation to its behaviour or physiology. The male had elevated expression of several genes that matched hypothetical or otherwise unannotated proteins in the genomes of *A. mellifera*, *C. floridanus *and *S. invicta*. It is particularly interesting that the male received so many fully unannotated protein matches (n = 851 contigs). This may suggest possible novel expression associates with male-specific behaviour and/or physiology. Thus, these data offer valuable insights into the mechanistic basis of male biology in social insects, which has largely been ignored by previous studies (see references above).

### The immune response in males

Even in the absence of overt infectious challenge, the background level of immune defence is likely to be regulated through the bumblebee life cycle. We did not explicitly challenge the sampled bees with pathogens, but also did not keep them in germ-free environments (pre-adults in their natal colony, adults in nurseries), and thus we expect a background level of immune activation. *B. terrestris *queens are monandrous, mating only once [65], while males can mate up to eight times [66]. In addition, *B. terrestris *exhibits highly male-biased sex ratios [67]. Together, this suggests high levels of competition among the males to mate with gynes. Consequently, males should invest in reproductive fitness, which has been demonstrated in other insect species to trade-off against immunity (e.g. *Anopheles gambiae *[68]). We identified genes involved in pathogen recognition, the transduction of recognition signals, and immune effectors, and analysed their patterns of expression for data to support this hypothesis. Surprisingly, in contrast to our expectations based on life-history theory, the male had elevated expression (compared to other stages) of AMPs involved in the removal of infectious agents as part of the immune system [69-72], including hymenoptaecin, defensin, abaecin and apidaecin. This is the first account of an apidaecin-type protein being expressed in *B. terrestris*. Wilfert et al. [73] found no trade-offs between either branch of the immune system (prophenoloxidase (PPO) and AMP) and reproductive investment, but rather a positive correlation between AMPs and reproduction. Wilfert et al. [73] suggest the basis for the positive correlation may be because males pass on AMPs with their sperm to mates during copulation. However, the male in our study was very young and we sampled the whole body rather than just reproductive tissue, making the mating-gift hypothesis less convincing. Unlike the majority of social insect males, bumblebee males do not remain within the colony post-emergence from the pupal case. Bumblebee males forage for themselves and spend the majority of their time patrolling scent-marked trails [26]. Bumblebee males can survive outside the colony for up to 60 days (Brown, M.J.F., unpublished data). Thus males cannot take advantage of proposed colony-level social immunity [74] and a primed immune system might be an adaptation to life outside the colony. Thus, our results suggest that the life-history differences between males (effectively solitary) and females (colonial) may impose divergent selection on expression of immune system genes in social insects.

### Olfaction in the bumblebee

Olfaction and the ability to discriminate a number of volatiles is of immense importance to insects in general, and social insects in particular. They must recognise nest-mates, discriminate and control subordinates, select mates, and discriminate between a wide range of plants for food collection. Here we discuss two classes of olfaction genes, the odorant binding proteins (OBPs) and the chemosensory proteins (CSPs).

In the honeybee genome, 21 OBP genes have been identified and examined for patterns of expression [75]. Within our transcriptome dataset, we found significant matches for eight *A. mellifera *OBPs (OBP1, OBP2, OBP3, OBP4, OBP6, OBP9, OBP11 and OBP13). This enables us to compare their expression in bumblebees to that of their homologues in honeybees. In the honeybee, OBP1, OBP4, OBP6 and OBP11 are expressed exclusively in the antennae of adults [75] and OBP11 was identified as having gender-specific expression (absent from honeybee drones), suggesting a role in female recognition of mates [75]. We found low expression of a *B. terrestris *orthologue of *A. mellifera *OBP1 (formerly known as antennal specific protein 1), which is involved in binding of queen pheromone in honeybees [76]. In honeybees, OBP1 is expressed in workers' and drones' antennae after approximately 14 days post-emergence [76]. As we sampled our bees before this late timepoint, level of expression of the OBP1 homologue may simply be due to developmental staging. OBP6 was expressed in all the adult bumblebee stages, but not the larva and pupa, matching expression patterns in the honeybee. In contrast, OBP11 was expressed in only the male and gyne. Consequently, OBP11 in the bumblebee may have a role in mate recognition within both sexes, as opposed to the putative female-specific role in honeybees.

OBP2 had elevated expression in all adult stages of *B. terrestris*. In honeybees, OBP2 is expressed specifically in the antenna with weak expression in the legs and head, possibly from chemosensory sensilla in these body parts [75]. In contrast, OBP3 is ubiquitously expressed in all adult body parts of the honeybee with the exception of the antennae [75]. In *B. terrestris*, OBP3 was highly expressed in all the bumblebee adult stages, with higher expression in the gyne than in the worker, but was absent from the larva and pupa, which again matches honeybee expression patterns. OBP9 is only expressed in the ovaries and eggs of the queen in honeybees [75] but in *B. terrestris *the male was the only sample to show expression of OBP9. Lastly, OBP13 was highly expressed in the *B. terrestris *pupa and male, but Forêt and Maleszka [75] identified expression of *A. mellifera *OBP13 in old larvae, with expression continued throughout the pupal stage. It appears that expression of these odorant binding proteins has been conserved in some cases, whilst diverging in others, presumably in response to taxon-specific selection processes.

We identified four putative CSPs (homologues of CSP2, CSP3, CSP4 and CSP6 in *A. mellifera*) in the *B. terrestris *transcriptome. *B. terrestris *CSP3 and CSP4 had elevated expression in the larva, matching the results of Forêt et al. [77] who found *A. mellifera *CSP3 to exhibit highest expression in the larva before pupal and imaginal moults. While Briand et al. [78] proposed that CSP3 had a role in brood pheromone recognition, Forêt et al. [77] proposed that CSP3 may play a role in cuticle maturation. In *A. mellifera*, CSP4 expression was restricted to olfactory tissues in adult, but not pre-adult stages [77]. In contrast, while we detected CSP4 expression in adult *B. terrestris*, the highest expression was in the larva. In honeybees, CSP2 was expressed at low levels throughout the life cycle stages while CSP6 was expressed throughout the larva, pupa and adult stages, with elevated tissue-specific expression in queen ovaries and eggs [77]. The expression of CSP2 and CSP6 across the bumblebee life stages is consistent with that of the honeybee. We were surprised not to have detected a homologue of *A. mellifera *CSP1, which is expressed ubiquitously across honeybee life cycle stages. Finally, *A. mellifera *CSP5 is expressed only by mature queen honeybees in eggs and ovaries, stages we did not sample in *B. terrestris*.

Overall, the examination of olfaction-related genes in *B. terrestris *reveals both similarities and also striking differences from their expression in *A. mellifera*. We expect that these divergences reflect the different social structuring of bumblebee compared to honeybee colonies, and in particular the differing roles and strategies adopted by different castes in these two species. Again, our results provide indications of fruitful lines of future research into how patterns of gene expression relate to the evolution of primitive (bumblebees) and advanced (honeybees) sociality.

## Conclusions

The role of differential gene expression in polyphenism within groups is only beginning to be understood, e.g. [4,11-15,79]. As a step towards understanding genes influential in the expression of specific phenotypes throughout an individual's life cycle stage, we performed a transcriptome-wide analysis of individual specimens of the pre-adult stages, castes and sexes of the bumblebee to improve our knowledge of differential gene expression across the life history of this ecologically and agriculturally important pollinator. We have identified sets of genes that are candidates for regulators and effectors of different phenotypes, and revealed the differing physiological states of each morph. These candidate genes will prove important for future genomic and proteomic studies on *B. terrestris*.

The *B. terrestris *genome is being sequenced, and a BAC library and a number of genetic linkage maps have been constructed to provide greater genomic resources for this important insect [73,80-82]. Our study contributes to this global effort through 504 Mb of Roche 454 transcriptomic data for *B. terrestris*. We provide a central, web-based transcriptomics resource for *B. terrestris*[83], that facilitates user querying of sequences and functional annotations. These data are now a bridgehead into deeper, molecular analyses of the physiological, genetic and evolutionary bases of polyphenism, and the further development of the bumblebee as a model social insect.

## Methods

### Animals

*B. terrestris *colonies (Koppert, Netherlands) were maintained at 27 ± 1°C (45-50% RH) under red light. Pollen and sugar water (ApiInvert^®^) were supplied to the colonies ad libitum. Two colonies were chosen where the founding queen was present, only worker callows were emerging from hatching pupal cases and sexual offspring, i.e., males and gynes, were absent. From one colony, individual specimens of worker life stages (larva, pupa and worker, known hereafter as worker 2) were collected. Adult stages (male, gyne and worker, known hereafter as worker 1) were similarly collected from the second colony. Workers were collected from both colonies to provide a comparison of gene expression across the colonies for the worker phenotype. In total, six individuals (one worker larva, one worker pupa, one male, one gyne and two worker adults) were collected for transcriptomic analyses. The larva and pupa were maintained in their natal colony and monitored daily using photography. At seven days of development, the larva (third instar) and pupa were removed, snap frozen in liquid nitrogen and stored at -80°C. Upon emergence, adults were removed to a nursery, which housed three distinguishable older workers, each with clipped wings, a brood clump, pollen and sugar water (providing both a normal social environment to stimulate normal gene expression in the newly emerged adults and a setting that was simple to monitor). The presence of older workers within the nursery would suppress ovarian development and subsequent egg laying within subordinate younger adults. Adults matured in the nursery for three days. Adults were then sacrificed by snap freezing in liquid nitrogen and stored at -80°C.

### RNA Extraction, cDNA synthesis and EST sequencing

Total RNA was isolated from the whole bodies of specimens using TRIzol Reagent (Invitrogen, UK) according to the manufacturer's instructions. Each specimen was ground using a high performance disperser (T-18 Basic ULTRA TURRAX, IKA^®^) in 5 ml of TRIzol reagent. 1.5 ml of chloroform (Sigma, Ireland) was added to the TRIzol extract, mixed by inversion and incubated at room temperature for 3 min. The sample was centrifuged at 11,500 g at 4°C for 15 min. The aqueous phase containing RNA was transferred to a fresh tube and an equal volume of 2-propanol (Sigma, Ireland) was added. The sample was vortexed, incubated at room temperature for ten min and then centrifuged at 11,500 g at 4°C for 10 min, resulting in the precipitation of the RNA out of solution in the form of a pellet. The supernatant was then removed, 70% ETOH was added, and the sample was vortexed and centrifuged at 9,250 g at 4°C for 5 min. The new supernatant was removed and the pellet was allowed to dry at room temperature. The final pellet was resuspended in 100 μl Elution Solution supplied with the GenElute™ Mammalian Total RNA kit (Sigma, Ireland), and RNA purified using the kit following the manufacturer's instructions. DNase treatment was performed using an On-column DNase (Sigma, Ireland) at a concentration of 70 μl of DNase Digest Buffer for On-column DNase Digestion to 10 μl of DNase I. Total RNA was quantified and integrity assessed on an Agilent 2100 Bioanalyzer using the Agilent RNA 6000 Nano Chip kit. cDNA was synthesised for 3 μg of RNA using the Evrogen SMART cDNA synthesis service (Evrogen, Russia). 5 μg of cDNA from each sample was used to create a Roche 454 sequencing library and sequenced on a Roche 454 Life Sciences GS FLX Titanium Series sequencer. The six *B. terrestris *samples were sequenced independently (one sample per lane), and base calling and quality screening performed using standard Roche pipeline version 2.3. Roche 454 sff files were deposited in the SRA on the EBI, library ERP000936.

### Transcriptome assembly

Roche 454 reads from the individual life cycle stages were combined to the purpose of assembly. We assembled the reads into a first draft transcriptome for *B. terrestris *following the approach of Kumar and Blaxter [41]. Two distinct assemblers were used to generate primary assemblies: gsAssembler from Roche/454 Life Sciences (also known as Newbler, version 2.5.3; with settings "-urt", "-cDNA", "-vt SMARTAdaptors.fna") (the Newbler_454 assembly), and MIRA (version 3.0.2;[84,85] (the MIRA_454 assembly). For MIRA assembly (settings: "--job=denovo,est,normal, 454"), SMART adaptors were removed using BLAST and a custom perl script.

The *B. terrestris *Sanger-sequenced expressed sequence tag (EST) data (generated by Sadd et al. [31]) and other pre-existing *B. terrestris *mRNAs in EMBL/GenBank were downloaded from EMBL nucleotide database (18th August 2010), and assembled using PartiGene [86](the PG_Sanger assembly).

We then coassembled the three contig sets (PG_Sanger, Newbler_454 and MIRA_454) using CAP3 [87](with settings: sequence similarity 98% and base overlap 50 bp), to generate BT_transcriptome_v1 contigs. Examination of the BT_transcriptome_v1 contigs both affirmed that the assembly generated contig sequences of higher credibility than from the single assemblers, but also identified issues of remaining redundancy in the data. We therefore reclustered the BT_transcriptome_v1 contig set using PartiGene to generate the final *B. terrestris *transcriptome assembly, BT_transcriptome_v2, for annotation and analysis. We mapped each of the input reads in the Roche 454 data back to this final BT_transcriptome_v2 assembly using BLAST and assessed stage-specific expression of each contig by counting reads per contig for each source library using a custom Perl script. The vast majority of the total reads (1,441,743 ESTs or 92.4%) were mapped to 33,875 BT_transcriptome_v2 contigs.

### Assessment of completeness and integrity of the assembly

We assessed the quality of BT_transcriptome_v2 by: (a) assessing congruence with available *B. terrestris *transcriptome shotgun assembly data from [32] (INDC Accession numbers: JI045408-JI025924.1) using BLAST [88]; and (b) assessing completeness compared to the whole-genome derived transcriptomes for other Hymenoptera (the official gene set (OGS) for the honeybee *A. mellifera *(from [89]), and the ants *C. floridanus *[NCBI Genome Project ID: 50201] and *H. saltator *[NCBI Genome Project ID: 50203]) and other arthropods (the OGS for *D. melanogaster *from the latest genome release [90]), using tBLASTx with an E-value cutoff of 1e-10.

### Functional Annotation

We annotated the BT_transcriptome_v2 contigs with best BLAST matches by comparing them to the NCBI nr database (27th March 2011; reporting up to five matches with an E-value cutoff of 1e-06). BT_transcriptome_v2 contigs were also annotated with Gene Ontology (GO), Enzyme Commission (EC) and Kyoto Encyclopaedia of Genes and Genomes pathways (KEGG) identifiers using annot8r (v.1.1.1;[91,92]) with a cut-off bit score of 55. GO terms were further summarised using the GO-Slim hierarchy. InterProScans were performed to infer putative function for hypothetical and unannotated contigs using Blast2GO software tool (V.2.4.8;[93-95]).

The annotated data were searched for genes, pathways and processes of interest. Gene lists were generated based on annotated processes and pathways for the eusocial honeybee, *A. mellifera*, the parasitoid jewel wasp, *N*. *vitripennis*, the holometabolous fruit fly, *D. melanogaster*, and the hemimetabolous pea aphid, *A. pisum*. Protein and nucleotide sequences of genes of interest were obtained from NCBI and compared to the *B. terrestris *transcriptome data using BLAST. Gene names and accession numbers used for these comparisons are given in Additional file 4.

### Comparison of expression between life stages of *B. terrestris*

R-STAT analysis devised by Stekel et al. [42], was used to identify contigs in BT_transcriptome_v2 that varied in their proportional contribution from each of the six sequenced samples. R-STAT calculates a log likelihood ratio statistic that estimates variation of proportional contribution to each contig from each sample. The resulting statistic, the R-value, expresses the variation in read contribution to each contig across life cycle stages. We treat the results from this study as preliminary because, given the absence of replication within life cycle stages and castes, these categories are confounded with the age of the individual specimens. Statistical analyses were carried out using the R language (version 2.11.1;[96]).

## Authors' contributions

TJC collected samples, isolated RNA and conducted the bioinformatics analysis of the contig set. JCC, SJB and MLB were involved in assembly and annotation of the contig set. The study was conceived and designed by SS, MLB and MJFB. All authors were involved in the writing of the manuscript. All authors have read and approved the final manuscript.

## Supplementary Material

Additional file 1 **Assembly pipeline of *Bombus terrestris *transcriptome**. This excel file contains all information regarding raw read data, primary assemblies and secondary assemblies for generation of BT_transcriptome_v2. Click here for file

Additional file 2 **Credibility testing of 454 versus Sanger data**. This pdf data contains information on proportion of secondary assembly generated from 454 and/or Sanger data and in addition, credibility tests on unassembled Sanger data. Click here for file

Additional file 3 **GoSlim analyses of BT_transcriptome_v2**. This excel file contains GO terms mapped to the BT_transcriptome_v2 contig set with individual pie-charts displaying contigs annotated with molecular function (MF), biological processes (BP) and cellular component (CC) ontologies. Click here for file

Additional file 4 **Gene sets for pathways and processes across the insect groups**. This excel file contains gene sets of peptide sequences generated for *A. mellifera*, *N. vitripennis*, *D. melanogaster *and *A. pisum*. Click here for file

Additional file 5 **Singletons and contigs receiving unique EST contribution from a life cycle stage**. This excel file contains information on number of unique singletons (sequences with one 454 read mapped), number of unique contigs (sequences with more than one 454 read mapped), number of ESTs contributed per life cycle stage to unique contig, total number of ESTs per life cycle stage, percentage of total ESTs from each life cycle stage contributed to unique contigs and percentage of total contig set for each group of stage-restricted contigs. Click here for file

Additional file 6 **Elevated expression within the individual life cycle stages**. This pdf contains information on the top ten highly expressed contigs that best distinguish a life cycle stage. Click here for file
