# Supplementary material for: Polyphenism in social insects: insights from a transcriptome-wide analysis of gene expression in the life stages of the key pollinator, Bombus terrestris
Source: BMC Genomics. 2011 Dec 20;12:623. doi: 10.1186/1471-2164-12-623 (PMC3276680; doi:10.1186/1471-2164-12-623)
Supplement: Additional file 2 — Credibility testing of 454 versus Sanger data. This pdf data contains information on proportion of secondary assembly generated from 454 and/or Sanger data and in addition, credibility tests on unassembled Sanger data. [file 1471-2164-12-623-S2.PDF]

Additional file 2:

Comparison of 454 and Sanger *B. terrestris* ESTs

The purpose of the 454 and Sanger comparison was to identify:

(a) novel data within the 454 dataset;

(b) identify biological credibility of unassembled secondary assembly singletons from PG_Sanger dataset.

Putative Genes within the 454 dataset

Of the 22,318 CAP3 secondary contigs, 14,675 were coassembled from the MIRA_454 and Newbler_454 primary assemblies only i.e. PG_Sanger-free CAP3 contigs (Table S2.1). A further 1,845 CAP3 secondary contigs were assembled by only one of the assemblers (Newbler_454: 138; and MIRA_454: 1,707).

Table S2.1. BT_transcriptome_v1 assembly.

Contribution from primary assembly contig set	Number of contigs
Assembly from all three primary assemblies	4,867
Assembly from two primary assemblies	
MIRA_454 plus PG_Sanger	414
Newbler_454 and PG_Sanger	500
MIRA_454 and Newbler_454	14,675
Assembly from only one primary assembly	
PG_Sanger contigs	17
MIRA_454 contigs	1,707
Newbler_454 contigs	138
Total contigs in CAP3 assembly	22,318

Number of contigs contributed from the respective primary assemblies to CAP3 secondary assembly.

CAP3 assembly resulted in the generation of 33,527 second order singletons, of which, 5,266 secondary assembly singletons consisted of PG_Sanger data (Table S2.2).

Table S2.2: Unassembled contigs within BT_transcriptome_v1 contig set.

Primary Assembly	Number of singletons
PG_Sanger	5266
MIRA_454	20835
Newbler_454	7512
Total	33527

Numbers of second order "singletons" (contigs from primary assembly not assembled by the CAP3 secondary assembly) per primary assembly within the CAP3 secondary.

Biological credibility of unassembled PG_Sanger contigs

To assess biological credibility of PG_Sanger contigs unassembled with the 454 data, the following parameters were examined:

(a) Length

Shorter sequences may result in non-assembly. CAP3 secondary contigs were on average longer in length than the unassembled PG_Sanger contigs. In addition, the N50 contig size was smaller for the PG_Sanger set (Table S2.3).

Table S2.3: Length comparison between CAP3 contigs and unassembled PG_Sanger contigs.

	CAP3 contigs	Unassembled PG_Sanger contigs
Mean contig length	1293.89	713.94
Max contig length	26,105	2,347
Min contig length	53	102
N50 contig size	1,911	701
Total number of contigs	22,318	5,266

Statistical information on comparisons of length, including mean contig length, max. contig length, minimum contig length and N50 contig size, of CAP3 secondary contigs and PG Sanger CAP3 secondary singletons.

(b) AT-richness

A higher AT-richness would reflect contamination by genomic DNA in comparison to cDNA and result in non-assembly of PG_Sanger contigs. AT richness calculations were equivalent for both contig sets at approximately 64%. The GC content (36%) of the content was expected.

(c) Number of singletons (i.e. sequences comprising 1 EST only) within the unassembled PG_Sanger contig set

As singletons from the primary assembly, the sequences may be poor quality and may be a possible reason for non-assembly in the CAP3 secondary assembly. Of the 5,266 PG_Sanger second order singletons, 4,091 were Sanger singletons (77.68%).

(d) BLAST matches against nr.

Sequences of low quality would be predicted to have no BLAST matches. Over half of the PG_Sanger second order singletons (n=2,966; 56%) did not return BLAST matches. While sequences not returning BLAST matches may consist of untranslated region, non coding RNA or potentially novel *Bombus* sequences, some may be sequencing artefacts and therefore, did not assemble with the 454 data.
