# Supplementary material for: Polyphenism in social insects: insights from a transcriptome-wide analysis of gene expression in the life stages of the key pollinator, Bombus terrestris
Source: BMC Genomics. 2011 Dec 20;12:623. doi: 10.1186/1471-2164-12-623 (PMC3276680; doi:10.1186/1471-2164-12-623)
Supplement: Additional file 6 — Elevated expression within the individual life cycle stages. This pdf contains information on the top ten highly expressed contigs that best distinguish a life cycle stage. [file 1471-2164-12-623-S6.PDF]

Additional file 6: Elevated expression within the individual life cycle stages.

Table S6a: Potential higher expression within *B. terrestris* larva.

BTT_contig	Functional description	Best BLAST match and GenBank accession [species]		L[a]	P[b]	W1[c]	W2[d]	M[e]	G[f]	Total[g]	R-value
BTT05364_1	Unknown	PREDICTED: similar to CG4409-PA [*A. mellifera*]	XP_001123230.1	5518	0	0	0	0	1	5520	8977
BTT03121_2	Endocuticle structural glycoprotein	Endocuticle structural glycoprotein SgAbd-1 [*C. floridanus*]	EFN60841.1	5208	3	0	0	0	0	5211	8466
BTT05366_1	Cuticular protein	PREDICTED: similar to CG7658-PA [*A. mellifera*]	XP_001120498.1	2153	0	0	0	0	0	2153	3489
BTT18911_1	Hexamerin	Hexamerin 70b [*A. mellifera*]	NP_001011600.1	2947	15	337	88	99	177	3663	3289
BTT20843_1	Cuticular protein	PREDICTED: similar to CG30045-PA [*A. mellifera*]	XP_001120541.1	1355	0	0	0	0	0	1355	2186
BTT12993_1	Carbonic anhydrase	Hypothetical protein SINV_01281 [*S. invicta*]	EFZ18639.1	1732	8	134	49	252	30	2205	1937
BTT14791_1	Hypothetical with no predicted IPR domains	PREDICTED: hypothetical protein [*A. mellifera*]	XP_001120555.1	1125	1	0	0	0	0	1126	1806
BTT35627_1	Hypothetical protein with IPR predicted transmembrane and signal peptide domains	PREDICTED: hypothetical protein [*A. mellifera*]	XP_001120555.1	984	2	0	0	0	0	986	1572
BTT20746_1	Cuticular protein	PREDICTED: similar to CG32405-PA	XP_001121128.1	951	0	0	0	0	0	951	1527

		[*A. mellifera*]									
BTT20966_1	Cytochrome P450	PREDICTED: similar to Probable cytochrome P450 6a14 (CYPVIA14) [*A. mellifera*]	XP_623362.2	889	366	0	0	0	0	1255	1451

L= Larva; P= Pupa; W1=Worker1; W2=Worker2; M=Male; G=Gyne; a= Total number of larva ESTs (n=286501); b= Total number of pupa ESTs (n=162608); c= Total number of worker1 ESTs (n=318664); d= Total number of worker2 ESTs (n=194969); e= Total number of male ESTs (n=257318); f= Total number of gyne ESTs (n=221683); g= Total number of ESTs (n=1441743).

Top ten BT_transcriptome_v2 contigs with EST-bias from larval sample with corresponding R-value and also putative nr BLAST description. Contribution of ESTs from other samples are provided.

Table S6b: Potential higher expression within *B. terrestris* pupa.

BTT_contig	Functional description	Best BLAST match and [species]	GenBank accession	L[a]	P[b]	W1[c]	W2[d]	M[e]	G[f]	Total[g]	R-value
BTT05433_2	Hexamerin	Hexamerin 110 [*A. mellifera*]	ABU92559.1	283	3384	0	0	0	0	3667	6628
BTT05434_1	Dorsal/ventral axon guidance	PREDICTED: similar to CG1845-PA [*A. mellifera*]	XP_395348.2	34	2638	38	21	26	38	2795	5011
BTT05442_1	Hexamerin	Hexamerin 110 [*A. mellifera*]	NP_001094493.1	119	2428	0	0	1	1	2549	4834
BTT05434_2	Beta-ureidopropionase	PREDICTED: similar to beta-ureidopropionase Isoform 1 [*A. mellifera*]	XP_392773.2	17	2489	34	20	32	18	2610	4805
BTT35255_1	Hexamerin	Hexamerin 110 [*A. mellifera*]	ABU92559.1	346	2376	0	0	0	0	2722	4551
BTT20945_1	Hexamerin	Hexamerin 110 [*A. mellifera*]	BAI82215.1	147	2304	0	1	0	0	2452	4547
BTT17751_1	Short-chain dehydrogenase /reductase	Short-chain dehydrogenase/reductase [*A. mellifera*]	NP_001011620.1	0	1760	0	0	0	0	1760	3708
BTT23482_1	Hexamerin	Hexamerin [*A. mellifera*]	ABR45905.1	1	1633	0	1	0	1	1636	3423
BTT35822_1	Hexamerin	Hexamerin [*A. mellifera*]	ABR45905.1	0	1585	0	0	0	0	1585	3337
BTT07410_1	Vitellogenin	PREDICTED: Hypothetical protein [*A. mellifera*]	XP_001121939.1	0	1512	0	0	0	1	1513	3177

L= Larva; P= Pupa; W1=Worker1; W2=Worker2; M=Male; G=Gyne; a= Total number of larva ESTs (n=286501); b= Total number of pupa ESTs (n=162608); c= Total number of worker1 ESTs (n=318664); d= Total number of worker2 ESTs (n=194969); e= Total number of male ESTs (n=257318); f= Total number of gyne ESTs (n=221683); g= Total number of ESTs (n=1441743).

Top ten BT_transcriptome_v2 contigs with EST-bias from pupal sample with corresponding R-value and also putative nr BLAST description. Contribution of ESTs from other samples are provided.

Table S6c: Potential higher expression within *B. terrestris* worker.

BTT_contig	Functional description	Best BLAST match and [species]	GenBank accession	L[a]	P[b]	W1[c]	W2[d]	M[e]	G[f]	Total[g]	R-value
BTT33135_1	Cytochrome P450	Cytochrome P450 4G11 [*A. mellifera*]	NP_001035323.1	84	10	1990	1472	541	827	4924	1944
BTT05272_1	Haemolymph juvenile hormone binding protein/allergen	PREDICTED: similar to CG3246-PA [*A. mellifera*]	XP_395658.3	7	41	1792	871	1453	1399	5563	1916
BTT22253_1	Cytochrome P450	Cytochrome P450 4G11 [*A. mellifera*]	NP_001035323.1	76	18	1361	1155	259	581	3450	1442
BTT20743_1	Hypothetical that contains a fibronectin type 1-like domain	Hypothetical protein EAG_09691 [*C. floridanus*]	EFN63274.1	0	2	1377	378	118	360	2235	1376
BTT00029_1	Glyceraldehyde-3-phosphate dehydrogenase	Hypothetical protein SINV_01281 [*S. invicta*]	EFZ17694.1	978	129	2842	1037	1837	2212	9035	1301
BTT24074_1	Cytochrome P450	Cytochrome P450 4G11 [*A. mellifera*]	NP_001035323.1	36	9	869	602	267	402	2185	793
BTT05313_2	Peritrophin-like proteins	PREDICTED: similar to CG10154-PA [*A. mellifera*]	XP_001119969.1	1	0	553	461	513	492	2020	753
BTT20590_1	Glucose oxidase	Glucose oxidase [*A. mellifera*]	NP_001011574.1	0	0	795	292	221	225	1533	724
BTT15820_1	Lipase	Lipase [*B. ignitus*]	ABY84699.1	7	1	488	425	91	43	1055	667
BTT05577_1	Hypothetical with no IPR predicted domains	PREDICTED: Hypothetical protein [*A. mellifera*]	XP_001121174.1	69	19	758	423	754	294	2317	651

L= Larva; P= Pupa; W1=Worker1; W2=Worker2; M=Male; G=Gyne; a= Total number of larva ESTs (n=286501); b= Total number of pupa ESTs (n=162608); c= Total number of worker1 ESTs (n=318664); d= Total number of worker2 ESTs (n=194969); e= Total number of male ESTs (n=257318); f= Total number of gyne ESTs (n=221683); g= Total number of ESTs (n=1441743).

Top ten BT_transcriptome_v2 contigs with EST-bias from worker 1 sample with corresponding R-value and also putative nr BLAST description. Contribution of ESTs from other samples are provided.

Table S6d: Potential higher expression within *B. terrestris* worker.

BTT_Contig	Functional description	Best BLAST match and [species]	GenBank accession	L[a]	P[b]	W1[c]	W2[d]	M[e]	G[f]	Total[g]	R-value
BTT05460_1	Alpha-glucosidase	Alpha-glucosidase [*B. ignitus*]	BAI44030.1	110	6	2677	6080	27	3	8903	10101
BTT20899_3	Sallimus	PREDICTED: similar to sallimus CG1915-PC, isoform C [*A. mellifera*]	XP_001121572.1	7	0	1176	1922	16	305	3426	2960
BTT20391_1	Bombolitin	Bombolitin [*B. ignitus*]	ACY09649.1	5	1	1677	1691	9	837	4220	2944
BTT21092_1	Alpha-glucosidase	Alpha-glucosidase [*B. ignitus*]	BAI44030.1	31	1	766	1685	3	0	2486	2822
BTT05294_1	Cytochrome P450	Cytochrome P450 4g15 [*H. saltator*]	EFN85148.1	106	29	1568	2115	576	762	5156	2201
BTT22199_1	Cytochrome P450	Cytochrome P450 4G11 [*A. mellifera*]	NP_001035323.1	71	21	1299	1426	463	696	3976	1547
BTT05276_1	Allergen-related	Protein G12 [*H. saltator*]	EFN86980.1	1	0	224	952	239	40	1456	1294
BTT05480_1	Diapause-related	Diapause-related protein 41 [*B. ignitus*]	ABP97090.1	0	0	309	694	67	120	1190	913
BTT05263_2	Bombolitin	N/A	N/A	1	0	333	444	0	193	971	712
BTT35235_1	Peritrophin-like protein	PREDICTED: similar to CG10154-PA [*A. mellifera*]	XP_001119969.1	1	0	423	546	190	226	1386	664

L= Larva; P= Pupa; W1=Worker1; W2=Worker2; M=Male; G=Gyne; a= Total number of larva ESTs (n=286501); b= Total number of pupa ESTs (n=162608); c= Total number of worker1 ESTs (n=318664); d= Total number of worker2 ESTs (n=194969); e= Total number of male ESTs (n=257318); f= Total number of gyne ESTs (n=221683); g= Total number of ESTs (n=1441743).

Top ten BT_transcriptome_v2 contigs with EST-bias from worker 2 sample with corresponding R-value and also putative nr BLAST description. Contribution of ESTs from other samples are provided.

Table S6e: Potential higher expression within *B. terrestris* male.

BTT_contig	Functional description	Best BLAST match and GenBank accession [species]		L[a]	P[b]	W1[c]	W2[d]	M[e]	G[f]	Total[g]	R-value
BTT05276_2	Allergen-related	Protein G12 [*H. saltator*]	EFN86980.1	0	0	1948	819	2267	458	5492	2658
BTT24170_1	Hymenoptaecin	Hymenoptaecin [*B. ignitus*]	ACA04899.1	1	22	0	0	1345	1	1369	2226
BTT24170_2	Hymenoptaecin	Hymenoptaecin [*B. ignitus*]	ACA04899.1	0	15	1	0	1171	0	1187	1948
BTT06274_2	Sentrin-specific protease	Hypothetical protein SINV_12294 [*S. invicta*]	EFZ15577.1	26	9	4	16	1027	6	1088	1555
BTT36277_1	Hymenoptaecin	Hymenoptaecin [*B. ignitus*]	ACA04899.1	0	8	3	0	882	0	893	1460
BTT05775_1	Titin	Titin [*C. floridanus*]	EFN64029.1	0	7	5	1	715	5	733	1147
BTT05289_1	Peritrophin-1-like protein	PREDICTED: hypothetical protein, partial [*A. mellifera*]	XP_001121537.1	0	0	678	758	830	580	2846	1123
BTT05501_1	Serine carboxypeptidase	Hypothetical protein SINV_11635 [*S. invicta*]	EFZ19168.1	46	7	507	583	975	163	2281	971
BTT00570_1	Hypothetical that contains a fibronectin type 1-like domain	Hypothetical protein EAG_09691 [*C. floridanus*]	EFN63274.1	4	0	272	45	825	173	1319	911
BTT09205_1	Hypothetical with an IPR predicted transmembrane and signal peptide domains	Hypothetical protein EAG_15494 [*C. floridanus*]	EFN66147.1	7	2	3	4	447	2	465	689

L= Larva; P= Pupa; W1=Worker1; W2=Worker2; M=Male; G=Gyne; a= Total number of larva ESTs (n=286501); b= Total number of pupa ESTs (n=162608); c= Total number of worker1 ESTs (n=318664); d= Total number of worker2 ESTs (n=194969); e= Total number of male ESTs (n=257318); f= Total number of gyne ESTs (n=221683); g= Total number of ESTs (n=1441743).

Top ten BT_transcriptome_v2 contigs with EST-bias from male sample with corresponding R-value and also putative nr BLAST description. Contribution of ESTs from other samples are provided.

Table S6f: Potential higher expression within *B. terrestris* gyne.

BTT_contig	Functional description	Best BLAST match and GenBank accession [species]		L[a]	P[b]	W1[c]	W2[d]	M[e]	G[f]	Total[g]	R-value
BTT05275_2	Hexamerin	Hexamerin [*A. mellifera*]	ABR45904.1	41	45	434	16	55	4884	5475	7619
BTT05260_1	Arylphorin	Arylphorin subunit alpha [*H. saltator*]	EFN82403.1	7	11	89	1	14	1050	1172	1639
BTT36615_1	Hexamerin	Hexamerin [*A. mellifera*]	ABR45904.1	1	4	35	3	3	442	488	693
BTT05275_1	Hexamerin	Hexamerin [*A. mellifera*]	ABR45904.1	1	0	44	1	1	409	456	653
BTT07422_1	Hypothetical protein with an IPR predicted signal peptide domain	Hypothetical protein [*B. patagonicus*]	ADX36407.1	0	0	8	7	10	396	421	643
BTT00028_1	Acyl-CoA delta-9 desaturase	Acyl-CoA delta-9 desaturase [*B. terrestris*]	CAW34805.1	342	2	167	271	245	733	1760	541
BTT20597_1	Hypothetical protein with IPR predicted transmembrane, signal peptide and fibronectin-type I like domains	Hypothetical protein EAG_09691 [*C. floridanus*]	EFN63274.1	8	0	295	61	317	364	1045	459
BTT05277_1	Hexamerin	Hexamerin [*A. mellifera*]	ABR45904.1	0	0	21	2	3	281	307	445
BTT05289_2	Peritrophin-like protein	PREDICTED: Hypothetical protein [*A. mellifera*]	XP_001121537.1	0	1	274	298	175	306	1054	430
BTT18720_1	Crooked protein	PREDICTED: similar to CG17218-PA [*A. mellifera*]	XP_001120771.1	1	0	291	109	247	304	952	381

L= Larva; P= Pupa; W1=Worker1; W2=Worker2; M=Male; G=Gyne; a= Total number of larva ESTs (n=286501); b= Total number of pupa ESTs (n=162608); c= Total number of worker1 ESTs (n=318664); d= Total number of worker2 ESTs (n=194969); e= Total number of male ESTs (n=257318); f= Total number of gyne ESTs (n=221683); g= Total number of ESTs (n=1441743).

Top ten BT_transcriptome_v2 contigs with EST-bias from gyne sample with corresponding R-value and also putative nr BLAST description. Contribution of ESTs from other samples are provided.
